# 45,X/46,XY mosaicism: report on 14 patients from a Brazilian hospital. A retrospective study

**DOI:** 10.1590/1516-3180-2014-1326729

**Published:** 2014-09-02

**Authors:** Rafael Fabiano Machado Rosa, Willy Francisco Bartel D'Ecclesiis, Raquel Papandreus Dibbi, Rosana Cardoso Manique Rosa, Patrícia Trevisan, Carla Graziadio, Giorgio Adriano Paskulin, Paulo Ricardo Gazzola Zen

**Affiliations:** I PhD. Clinical Geneticist, Universidade Federal de Ciências da Saúde de Porto Alegre (UFCSPA) and Complexo Hospitalar Santa Casa de Porto Alegre (CHSCPA), Porto Alegre, Rio Grande do Sul, Brazil; II MD. Physician, Residency Program in Obstetrics and Gynecology, Hospital Materno Infantil Presidente Vargas (HMIPV), Porto Alegre, Rio Grande do Sul, Brazil; III MD. Postgraduate Student, Postgraduate Program in Pathology, Universidade Federal de Ciências da Saúde de Porto Alegre (UFCSPA), and Gynecologist and Obstetrician, Complexo Hospitalar Santa Casa de Porto Alegre (CHSCPA), Porto Alegre, Rio Grande do Sul, Brazil; IV MD. Postgraduate Student, Postgraduate Program in Pathology, Universidade Federal de Ciências da Saúde de Porto Alegre (UFCSPA), and Pediatrician, Grupo Hospitalar Conceição (GHC), Porto Alegre, Rio Grande do Sul, Brazil; V MD. Postgraduate Student, Postgraduate Program in Pathology, Universidade Federal de Ciências da Saúde de Porto Alegre (UFCSPA), and Pharmacist, Pontifícia Universidade Católica do Rio Grande do Sul (PUCRS), Porto Alegre, Rio Grande do Sul, Brazil; VI MD. Postgraduate Student, Postgraduate Program in Pathology and Professor of Clinical Genetics, Universidade Federal de Ciências da Saúde de Porto Alegre (UFCSPA), and Clinical Geneticist, Universidade Federal de Ciências da Saúde de Porto Alegre (UFCSPA) and Complexo Hospitalar Santa Casa de Porto Alegre (CHSCPA), Porto Alegre, Rio Grande do Sul, Brazil; VII PhD. Professor of Clinical Genetics and of the Postgraduate Program in Pathology, Universidade Federal de Ciências da Saúde de Porto Alegre (UFCSPA), and Clinical Geneticist, Universidade Federal de Ciências da Saúde de Porto Alegre (UFCSPA) and Complexo Hospitalar Santa Casa de Porto Alegre (CHSCPA), Porto Alegre, Rio Grande do Sul, Brazil

**Keywords:** Genitalia, Mosaicism, Turner syndrome, Azoospermia, Neoplasms, Genitália, Mosaicismo, Síndrome de Turner, Azoospermia, Neoplasias

## Abstract

**CONTEXT AND OBJECTIVE:**

45,X/46,XY mosaicism, or mixed gonadal dysgenesis, is considered to be a rare disorder of sex development. The aim of our study was to investigate the clinical and cytogenetic characteristics of patients with this mosaicism.

**DESIGN AND SETTING:**

A retrospective study in a referral hospital in southern Brazil.

**METHODS:**

Our sample consisted of patients diagnosed at the clinical genetics service of a referral hospital in southern Brazil, from 1975 to 2012. Clinical and cytogenetic data were collected from the medical records.

**RESULTS:**

Fourteen patients were included in the sample, with ages at the first evaluation ranging from 2 days to 38 years. Nine of them had female sex of rearing and five, male. Regarding the external genitalia, most were ambiguous (n = 10). One patient presented male phenotype and was treated for a history of azoospermia, while three patients presented female phenotype, of whom two had findings of Turner syndrome and one presented secondary amenorrhea alone. Some findings of Turner syndrome were observed even among patients with ambiguous genitalia. None presented gonadal malignancy. One patient underwent surgical correction for genital ambiguity and subsequent exchange of sex of rearing. Regarding cytogenetics, we did not observe any direct correlation between percentages of cell lines and phenotype.

**CONCLUSIONS:**

45,X/46,XY mosaicism can present with a wide variety of phenotypes resulting from the involvement of different aspects of the individual. All these observations have important implications for early recognition of these patients and their appropriate management.

## Abstract (pt)

**CONTEXTO E OBJETIVO:**

O mosaicismo 45,X/46,XY, ou disgenesia gonadal mista, é considerado uma doença rara do desenvolvimento sexual. O objetivo do nosso estudo foi verificar as características clínicas e citogenéticas de pacientes com este mosaicismo.

**TIPO DE ESTUDO E LOCAL:**

Estudo retrospectivo em um hospital de referência no sul do Brasil.

**MÉTODOS:**

Nossa amostra foi composta por pacientes diagnosticados em um serviço de genética clínica de um hospital de referência no sul do Brasil, no período de 1975 até 2012. Os dados clínicos e citogenéticos foram coletados a partir dos prontuários médicos.

**RESULTADOS:**

Catorze pacientes foram incluídos na amostra, idades na primeira avaliação variando de 2 dias a 38 anos. Nove deles apresentavam sexo feminino de criação e cinco, masculino. A genitália externa, na maioria, era ambígua (n = 10). O paciente com fenótipo masculino foi tratado por história de azoospermia, enquanto que das três pacientes do fenótipo feminino, duas apresentavam achados da síndrome de Turner e a outra, amenorreia secundária isolada. Alguns achados da síndrome de Turner foram observados mesmo entre pacientes com genitália ambígua. Nenhum deles apresentou neoplasia gonadal. Um paciente foi submetido à correção cirúrgica de ambiguidade genital e posterior troca de sexo de criação. Quanto à citogenética, não observamos correlação direta entre a porcentagem de linhas de células e o fenótipo.

**CONCLUSÕES:**

O mosaicismo 45,X/46,XY pode apresentar grande variedade de fenótipos resultantes do envolvimento de diferentes aspectos do indivíduo. Todas essas observações têm implicações importantes para o reconhecimento precoce desses pacientes e seu adequado manejo.

## INTRODUCTION

Disorders of sex development, previously called intersex disorders, are congenital conditions in which the development of chromosomal, gonadal or anatomical sex is atypical. The gonadal sex is determined by the structural, biochemical and functional characteristics of the gonad. The anatomical sex considers the physical appearance of the individual. The chromosomal sex is based on the result from karyotype analysis, and this is currently considered to be the main finding for classifying disorders of sex development. Among the broad variety of conditions that these disorders encompass is 45,X/46,XY mosaicism, also known as mixed gonadal dysgenesis.^1^ Loss of the Y chromosome due to non-disjunction subsequent to normal disomic fertilization is considered to be one hypothesis for the origin of this mosaicism.^2^

45,X/46,XY mosaicism is a rare and probably underdiagnosed condition, and its incidence is 1.5 per 10,000 newborns.^3 ^It is considered to be a phenotypically very heterogeneous condition. Although most of the cases diagnosed during the prenatal period are phenotypically normal males, attentions is usually drawn to cases identified after birth due to the presence of genital/gonadal anomalies.^2^ Thus, this great clinical variability may make it difficult to recognize individuals with 45,X/46,XY, which can lead to important implications for management of such cases, including the need to perform prophylactic gonadectomy due to tumor risk.

## OBJECTIVE

The aim of our study was to describe the experience of our service relating to 45,X/46,XY mosaicism, through investigation of the clinical and cytogenetic characteristics of individuals diagnosed over a period of 38 years.

## METHODS

The sample consisted of all patients diagnosed at the clinical genetics service of a referral hospital in southern Brazil, over the period from August 1975 to December 2012. The criterion for inclusion in the study was the presence of 45,X/46,XY mosaicism found through karyotype analysis on peripheral blood cells (lymphocytes). This examination was carried out in the university's cytogenetics laboratory through the technique of GTG banding.

We conducted a retrospective analysis by gathering clinical and cytogenetic data from the medical records. These data consisted of age at first evaluation, medical specialty that referred the patient, sex of rearing, characteristics of external and internal genitalia, and other major anomalies and dysmorphia observed in other organs and systems. A phenotype of ambiguous genitalia was considered to be present when the patient met the following criteria: 1) manifested genital ambiguity; 2) apparent female genitalia with an enlarged clitoris and labial fusion; and 3) apparent male genitalia with bilateral cryptorchidism, hypospadias or micropenis.^4^ The findings relating to the external and internal genitalia were described based on the masculinization score suggested by Ahmed et al.^5^ and applied by Cools et al.^6^

With regard to cytogenetic data, the number of metaphases analyzed and their proportions were ascertained in accordance with the 45,X and 46,XY cell lines, in peripheral blood and gonadal tissues. 

This project was approved by the Research Ethics Committees of the university and the hospital.

## RESULTS

Over the period studied, 14 patients with 45,X/46,XY mosaicism were identified. None of them had any description of associated structural abnormalities of the Y chromosome in the 46,XY cell line. Their ages at the first evaluation ranged from 2 days to 38 years (median of two years). Half of the cases (50%) were referred by Endocrinology, 35.8% by Pediatrics, 7.1% by Pediatric Surgery and 7.1% by Urology. Nine of them (64%) had female sex of rearing and five (36%), male. Regarding external genitalia, 71.5% (n = 10) were considered ambiguous. Among the remaining patients, 7.1% (n = 1) were male and 21.4% (n = 3) were female. The masculinization scores for the external genitalia presented by the patients are shown in Table 1. These ranged from 4 to 12 (maximum score) among the individuals with male sex of rearing and from 1 (minimum score) to 1.5 among the individuals with female sex of rearing.

**Table 1:** Clinical features involving the external and internal genitalia and cytogenetic findings presented by the patients

Features	Patients																	Total
	1	2	3	4	5		6		7	8	9	10	11	12	13		14	n = 14
Age at first evaluation	24 y	2 y	1 y	1 m	2 m		5 y		3 m	11 y	14 y	8 d	7 m	2 d	9 y		38 y	2 d to 38 y
Sex of rearing	M	M	M	M	M		F		F	F	F	F	F	F	F		F	5M and 9F
External genitalia^*^	M	A	A	A	A		A		A	A	A	A	A	F	F		F	1M-10A-3F
Scrotal fusion	+	+	+	+	+		-		-	-	-	-	-	-	-		-	5
Phallus (not micropenis)	+	+	+	-	-		-		-	-	-	-	-	-	-		-	3
Micropenis/clitoromegaly	-	-	-	+	+		+		+	+	+	+	+	-	-		-	8
Clitoris	-	-	-	-	-		-		-	-	-	-	-	+	+		+	3
Urethral meatus																		
Normal	+	-	-	-	-		-	-		-	-	-	-	-	-		-	1
Glandular	-	-	-	-	-		-	-		-	-	-	-	-	-		-	-
Penile	-	-	-	-	-		-	-		-	-	-	-	-	-		-	-
Perineal	-	+	+	+	+		+	+		+	+	+	+	+	+		+	13
Right and left gonads																		
Scrotal	+/+	+	-	-		-	-	-		-	-	-	-	-	-	-		2
Inguinal	-	-	-	-		-	+	-		-	-	-	-	-	-	-		1
Abdominal	-	+	+/+	+/+		+/+	+	+/+		+/+	+/+	+/+	+/+	+/+	+/+	+/+		13
Score of external genitalia^†^	12	8	7	4		4	1.5	1		1	1	1	1	1	1	1		1-12
Internal genitalia																		
Uterus	-	+	-	-		+	+	+		-	+	+	+	+	+	+		10
Inguinal hernia	-	-	-	+		-	-	-		-	-	-	+	-	+	-		3
Gonadectomy	-	+	-	+		+	-	+		-	-	+	-	+	-	-		6
Karyotype																		
45,X cells (%)	33	31	10	54		94	43	39		36	62	22	52	84	96	77		10-96
46,XY cells (%)	67	69	90	46		6	57	61		64	38	78	48	16	4	23		6-90

* Lee et al.4

† according to Ahmed et al.5

n = sample size

d = days

m = months

y = years

M = male

F = female

A = ambiguous

+ = present

- = absent.

The patient with a normal male phenotype (patient 1) was attended due to a history of infertility and azoospermia (he had a normal hormonal evaluation), while among the three patients with a female phenotype, two had findings of Turner syndrome (patients 12 and 13) and one presented secondary amenorrhea (patient 14). The last patient had reached menarche at the age of 15 and subsequently had only had three menstrual cycles. She did not present any report of short stature. The additional clinical abnormalities of the patients are outlined in Table 2. Some findings of Turner syndrome, such as short stature, widely spaced nipples and hypoplastic nails, were observed even among the patients with ambiguous genitalia. 

**Table 2:** Additional clinical features observed in the patients of the sample

Features	Patients														Total
	1	2	3	4	5	6	7	8	9	10	11	12	13	14	n = 14
Short stature	-	-	+	-	+	+	+	-	-	-	-	+	+	-	5
Obesity	-	-	-	-	-	-	-	-	-	-	-	-	+	-	1
Low posterior implantation of hair	-	-	-	-	-	+	-	-	-	-	-	-	-	-	1
Epicanthic folds	-	-	-	-	-	-	-	-	-	-	-	-	+	-	1
Nystagmus	-	-	+	-	-	-	-	-	-	-	-	-	-	-	1
Congenital glaucoma	-	-	+	-	-	-	-	-	-	-	-	-	-	-	1
High arched palate	-	-	-	-	-	-	-	-	-	-	+	-	-	-	1
Micrognathia	-	-	-	-	-	-	-	-	-	-	-	+	-	-	1
Posteriorly rotated ears	-	-	-	-	-	-	-	-	-	-	-	+	-	-	1
Short and webbed neck	-	-	-	-	-	-	-	-	-	-	-	+	-	-	1
Widely spaced nipples	-	-	-	-	-	+	-	-	-	-	-	+	+	-	3
Short and wide thorax	-	-	-	-	-	-	-	-	-	-	-	+	-	-	1
Sternal cutaneous appendix	-	-	-	-	-	-	-	-	-	-	-	+	-	-	1
Congenital heart defect	-	-	-	-	-	+	-	-	-	-	-	-	-	-	1
Renal anomaly	-	-	-	-	-	-	-	-	-	-	-	+	-	-	1
Cubitus valgus	-	-	-	-	-	+	-	-	-	-	-	-	-	-	1
Hypoplastic nails	-	-	-	+	-	-	-	-	-	-	+	+	+	-	4
Shortening of metatarsals	-	-	-	-	-	-	-	-	-	-	-	+	-	-	1
Lymphedema of hands and feet	-	-	-	-	-	-	-	-	-	-	-	-	+	-	1
Seizures	-	-	-	-	-	-	+	-	-	-	-	-	-	-	1
Neuropsychomotor delay	-	-	+	-	-	-	-	-	-	-	-	-	-	-	1
Hypothyroidism	-	-	-	-	-	-	-	-	+	-	-	-	-	-	1
Karyotype															
45,X cells (%)	33	31	10	54	94	43	39	36	62	22	52	84	96	77	10-96
46,XY cells (%)	67	69	90	46	6	57	61	64	38	78	48	16	4	23	6-90

+ = present

- = absent

Prophylactic gonadectomy, due to the risk of cancer, was described in six cases (42.9%). None of the patients presented any report of gonadal malignancy. Two patients with ambiguous genitalia were followed up until adulthood, and both presented amenorrhea (one underwent gonadectomy). One patient underwent exchange of sex of rearing (changed from male to female, patient 5 in Tables 1 and 2). 

Regarding cytogenetic analysis, 15 to 61 blood cells (mean of 36.7 cells) were analyzed per case for karyotyping. There was no correlation between the percentages of cell lines and the individual's phenotype. Variations were observed even among individuals who underwent gonadal karyotyping (Table 3).

**Table 3:** Percentages of 45,X and 46,XY cell lines presented by the patients who underwent karyotyping of peripheral blood and gonads

Patients	Karyotype cell line	Peripheral blood (%)	Right gonad (%)	Left gonad (%)
3	45,X	10	75	90
	46,XY	90	25	10
10	45,X	22	8	63
	46,XY	78	92	37

## DISCUSSION

In reviewing the literature, we found that only one study had been conducted in Latin America to evaluate the clinic and cytogenetic characteristics of patients with 45,X/46,XY mosaicism. This was developed by Rosenberg et al.,^7^ and evaluated the phenotypic findings of five patients. Other studies in the literature were developed in Europe^2^^,^ 8 and, especially, in the United States.^9^^-^ 13

Sexual differentiation is a complex process, and the *SRY* (sex-determining region Y) gene is critical within this cascade of events. Until the sixth week of gestation, the gonads of embryos of both sexes are indistinguishable. Individuals with a 46,XY chromosome constitution, through the effect of the *SRY *gene, will develop testes. These begin to be morphologically identified from the seventh to eighth week. In this process, the Sertoli cells are the first to become recognizable. These are organized as cells surrounding tubules and produce Müllerian inhibiting factor (MIF), a hormone that through local diffusion leads to regression of the Müllerian duct derivatives (which give rise to the uterus, uterine tubes and proximal vagina). Leydig cells produce an androgen, testosterone, which induces Wolff ducts to differentiate into epididymis, vas deferens, seminal vesicles and ejaculatory duct. In most distant urogenital tissues, such as the genital tubercle, conversion of testosterone into dihydrotestosterone by the enzyme 5-alpha-reductase is required. On the other hand, in an individual with a constitution without a Y chromosome, the gonad develops as an ovary, which makes the external genitalia remain female, the Müllerian ducts form the uterus and the uterine tubes, and the Wolff ducts regress. In the case of individuals with a 45,X chromosomal line, the lack of a second X chromosome lead to the development of streak gonads, because a second X chromosome is essential for full development and functioning of the ovaries.^8^

Mosaicism is defined as the presence of two or more chromosomal lineages in the same individual. In the case of 45,X/46,XY mosaicism, there is simultaneous presence of a lineage with X monosomy (45,X) and another with male constitution (46,XY). The different distributions of the 45,X and 46,XY chromosomal cell lines among the tissues in individuals with this mosaicism presumably reflect the wide variety of phenotypes observed, not only in our sample but also in the literature.^2^^,^ 7 ^-^ 13 Clinical variations have been observed even among monozygotic twins.^14^^,^ 15 In our study, the individuals with 45,X/46,XY mosaicism ranged from phenotypically normal men with azoospermia, going through individuals with genital ambiguity, to women with or without short stature and Turner syndrome.

45,X/46,XY mosaicism can be detected in individuals with normal male external genitalia. This phenotype is usually associated with bilateral testes^16^ and possible predominance of the 46,XY cell line in the gonads.^13^ In fact, this is considered to be the most common phenotype among patients with 45,X/46,XY mosaicism, since 89-95% of the individuals diagnosed during the prenatal period are male.^11^^,^ 17 Phenotypically normal males presenting infertility/azoospermia, as seen in our sample, have been reported among patients with 45,X/46,XY mosaicism only rarely.^2^^,^ 12 ^,^ 18 Moreover, despite also being uncommon, cases of 45,X/46,XY mosaicism have been reported among individuals with infertility. Penna Videaú et al.^19^ identified one case (1.2%) among 84 men with a history of infertility. Gassó-Matoses et al.,^20^ in turn, described an adult male with testes of smaller size and infertility. Interestingly, in this case the 46,XY cell line exhibited a structural abnormality consisting of a deletion on the long arm. Unlike our case (which was evaluated as hormonally normal), this patient had high levels of follicle-stimulating hormone. Structural alterations to the Y chromosome in the 46,XY cell line of the mosaic have been described, including involvement of the AZF regions. Interestingly, deletion of one or more loci in these regions can affect the development of the testes and cause impaired spermatogenesis.^21^ In our patient with azoospermia, we cannot rule out the possibility that a microscopic structural abnormality such as a microdeletion might exist, thereby affecting the AZF region. This would help to explain the findings relating to this patient.

Patients with male external genitalia can present hypospadias and/or cryptorchidism, as observed in four of our patients. However, it is noteworthy that among our cases, all the patients presented a severe form of hypospadias (all of them had perineal hypospadias). Aarskog^22^ found three cases of 45,X/46,XY mosaicism in a series of 100 boys with hypospadias. All of them were also associated with cryptorchidism (two unilateral and one bilateral), as also seen in our patients. Because these patients' testes are histologically closer to normality, they are often associated with Wolff duct derivatives. Such patients may present pubertal virilization and fertility, and the development of Müllerian derivatives is uncommon.^23^ However, some patients may present pubertal delay through failure of testicular development.^24^

Individuals with 45,X/46,XY mosaicism presenting male external genitalia may also exhibit short stature.^23^^-^ 26 This feature was observed in two patients of our sample, and both of them also presented hypospadias. The slower growth in these patients appears to be related to deficiencies of both their hypothalamic-gonadal axis and their primary gonadal function.^26^ In girls with short stature of unexplained cause, karyotyping is performed systematically, due to the possibility of a diagnosis of Turner syndrome. However, this procedure is not routinely followed for boys with short stature, especially if there are no other congenital anomalies, which may lead to underdiagnosis of 45,X/46,XY mosaicism. However, due to the low number of reports of phenotypically male individuals with 45,X/46,XY mosaicism, it is not possible to make any estimate of the percentage of cases presenting short stature.^27^ Furthermore, there have not been any studies evaluating the frequency of this mosaicism among males with short stature of unknown origin. Interestingly, these patients, just like those with Turner syndrome, would present benefits from therapy with growth hormones.^23^^,^ 25 ^,^ 26

In postnatal studies, the most common clinical presentation of 45,X/46,XY mosaicism is ambiguity of the external genitalia. This phenotype is usually associated with two dysgenetic testes or with a streak gonad and a contralateral dysgenetic testis.^16^ This postnatal frequency is probably due to bias, since these are the patients to whom attention is most drawn, because of the presence of genital abnormalities. In our study, 71.4% (n = 10) of the patients had ambiguous external genitalia.

The diagnostic investigation in these cases tends to start in the neonatal period. However, in our sample, it is noteworthy that five patients with genital ambiguity were referred for evaluation only after the first year of life (one of them came to be evaluated only at the age of 14 years), and thus their diagnoses were delayed. Furthermore, these patients were usually referred by specialized services, such as Endocrinology, which suggests that this mosaicism may go unnoticed by pediatricians, who are generally the first professionals to see these children. These points may have a direct influence on the management of these patients, including the question of choosing the sex of rearing. In our sample, there was a case in which the sex of rearing was changed later on, from male to female sex.

Some major anomalies such as cardiovascular and renal abnormalities, and dysmorphia such as low implantation of hair, shortening of metacarpal and metatarsal bones and a short and webbed neck, which are observed in individuals with Turner syndrome, have also been observed among patients with 45,X/46,XY mosaicism.^8^^,^ 9 ^,^ 28 This could be seen in our sample, as shown in Table 2. One of them had hands and feet presenting lymphedema. The presence of these findings suggests that this is an expression of a somatic 45,X cell line.

The phenotype of female external genitalia is usually associated with bilateral streak gonads.^16^ Individuals presenting 45,X/46,XY may have stigmata of Turner's syndrome without signs of virilization, and so may be clinically indistinguishable from those presenting 45,X.^29^ Out of all the patients diagnosed with Turner syndrome, about 2% to 5% are individuals with 45,X/46,XY.^29^^,^ 30 Stigmata and congenital anomalies associated with Turner syndrome are more common in children with 45,X/46,XY mosaicism presenting a female rather than a male phenotype.^13^ This may have a relationship with predominance of a 45,X cell line. These observations were evident in two patients of our sample (Table 2).

In our review of the literature, we found only one case similar to ours, in which a patient with 45,X/46,XY mosaicism with a normal female phenotype presented secondary amenorrhea. Jiao et al.^31^ identified one case among 531 Chinese patients with premature ovarian failure (0.2%). These authors explained the presence of menstruation in this case as possibly secondary to the expression of the 45,X cell line. Interestingly, although streak gonads are usually present in individuals presenting 45,X, about 3% of such adults spontaneously menstruate (at least twice) and 5% show breast development.^29^

The prevalence of tumors, especially gonadoblastomas and dysgerminomas, is greater among subjects with 45,X/46,XY mosaicism, regardless of their phenotype. According to Verp and Simpson,^16^ the prevalence of neoplasms in patients with this mosaicism appears to be about 15% to 20%, although it is possibly lower among patients with bilateral streak gonads or close-to-normal testes. However, some authors have suggested that these frequencies could be even lower.^32^ Tumor development also occurs more often the second decade of life. This may be the reason why we did not have any cases of tumors in our sample, since most of our patients consisted of children at their first evaluation and long-term monitoring was only achieved in a few cases. Furthermore, many patients underwent prophylactic gonadectomy (42.9%).

Although close monitoring of patients with 45,X/46,XY mosaicism is essential, there is unfortunately no consensus on what the best form of management should be. Cools et al.^6^ suggested that the masculinization score for the external genitalia could reflect both gonadal differentiation and tumor risk in patients with 45,X/46,XY mosaicism. They also suggested that in patients with a high degree of masculinization, orchidopexy should be performed in cases of cryptorchidism, and that self-examination should be performed regularly, along with annual ultrasound after puberty. Pre-pubertal and post-pubertal biopsies should be performed to assess the risk of tumors through specialized immunohistochemical analysis. Gonadectomy should be performed in cases of findings of pre-malignancy or cancer *in situ*. In cases of ambiguous genitalia, there should be a low threshold for performing gonadectomy. In cases of female phenotype, elective gonadectomy should be performed.^6^ The finding of early puberty and breast development in an individual presenting 45,X/46,XY should always raise the suspicion of malignancy.^16^

One of the most important and difficult points in treating patients with 45,X/46,XY mosaicism is the decision about the sex of rearing. Several factors are involved in this process, but the degree of virilization of the external genitalia and the presence of gonads with testicular features in the labioscrotal folds stand out among them. The parents' impression is another factor that should be taken into consideration. The risk of developing cancer and its management should also be assessed irrespective of the choice of gender. Thus, individuals who are well virilized and present gonads in the labioscrotal folds present the best conditions for choosing male sex of rearing.^8^^,^ 33

Unfortunately, however, there is no consensus on what would be the best way of handling these patients.^11^ Cools et al.^6^ stated that the masculinization score for the external genitalia that was suggested by Ahmed et al.^5^ could reflect both gonadal differentiation and the risk of tumors in patients with 45,X/46,XY mosaicism (malignancy risk seems to be inversely related to this score). 

We applied this tool to our sample. The patients with male sex of rearing had the highest scores, which ranged from 4 to 12 (maximum score). According to Cools et al.,^6^ patients with a score greater than or equal to 7, for their degree of virilization, are the ones who present the best conditions for choosing male sex of rearing. Interestingly, the patient who underwent exchange of sex of rearing (from male to female) had a score lower than 7 (the score was 4). Moreover, all the patients with female sex of rearing in our sample had scores of between 1 and 1.5. With regard to the internal genitalia, we evaluated the patients only for the presence or absence of a uterus. In cases with evidence of this internal organ, the choice of sex of rearing was usually female (except for patient 2, who had a score of 8, i.e. good virilization of the external genitalia; and initially for patient 5, who had a score of 4 and for whom, as mentioned earlier, a decision was made, later on, to change to female sex of rearing).

It is important to point out that patients with 45,X/46,XY mosaicism who undergo gonadectomy or present gonadal dysgenesis require continuous treatment with sex steroid hormones to promote the development of secondary sexual characteristics and prevent osteoporosis. Hormonal evaluation of patients with 45,X/46,XY mosaicism is performed similarly to that of patients with ambiguous genitalia, with doses of sexual hormones such as FSH, LH, testosterone and anti-Müllerian hormone.^4^

Our cytogenetic findings, in agreement with Telvi et al.,^2^ suggest that the phenotype of individuals with 45,X/46,XY mosaicism has no relationship with the proportion of 45,X cells, observed not only in lymphocytes but also in the gonads. The percentage of mosaicism found in amniotic fluid samples, in cases of prenatal diagnosis, is also a poor predictor of the phenotype.^11^ These difficulties makes genetic counseling a challenge during pregnancy. However, some authors have suggested that the proportions of 45,X and 46,XY cell lines found in gonads, rather than those in blood lymphocytes, would determine the degree of virilization.^13^

Our series with 45,X/46,XY mosaicism illustrates the importance of a multidisciplinary approach, involving several specialties such as Pediatrics, Clinical Genetics, Endocrinology, Pediatric Surgery and Psychology, in cases of disorders of sex development. Future reports, especially with long-term follow-up, will also be important, in order to better address some questions relating to management of such patients, such as tumor risk and sex of rearing. Another issue to be evaluated and not discussed in our series could be in relation to the gender identity and social behavior presented by these patients. It is known that the presence of a Y chromosome in brain tissue and subsequent prenatal exposure to androgens are major biological factors that could contribute towards the development of a male gender identity.^13^

## CONCLUSIONS

45,X/46,XY mosaicism can present with a wide variety of phenotypes resulting from involvement of different aspects of the individual, such as growth, hormone balance, gonadal development, sex of rearing and fertility. Children with this mosaicism also require a clinical evaluation similar to that conducted on girls with Turner syndrome, due to the possibility that, for example, associated cardiovascular and renal abnormalities might be present. All of these observations have important implications not only for early recognition of these patients but also for their proper management.
